# HERV-W ENV antigenemia and correlation of increased anti-SARS-CoV-2 immunoglobulin levels with post-COVID-19 symptoms

**DOI:** 10.3389/fimmu.2022.1020064

**Published:** 2022-10-27

**Authors:** Karen Giménez-Orenga, Justine Pierquin, Joanna Brunel, Benjamin Charvet, Eva Martín-Martínez, Hervé Perron, Elisa Oltra

**Affiliations:** ^1^ Escuela de Doctorado, Universidad Católica de Valencia San Vicente Mártir, Valencia, Spain; ^2^ Geneuro-Innovation, Bioparc Laënnec, Lyon, France; ^3^ National Health Service, Manises Hospital, Valencia, Spain; ^4^ GeNeuro, Geneva, Switzerland; ^5^ Department of Pathology, School of Health Sciences, Universidad Católica de Valencia San Vicente Mártir, Valencia, Spain; ^6^ Centro de Investigación Traslacional San Alberto Magno, Universidad Católica de Valencia San Vicente Mártir, Valencia, Spain

**Keywords:** HERV-W, long COVID-19, post-COVID, SARS-CoV-2, serology, IgE, chronic fatigue syndrome, CFS

## Abstract

Due to the wide scope and persistence of COVID-19´s pandemic, post-COVID-19 condition represents a post-viral syndrome of unprecedented dimensions. SARS-CoV-2, in line with other infectious agents, has the capacity to activate dormant human endogenous retroviral sequences ancestrally integrated in human genomes (HERVs). This activation was shown to relate to aggravated COVID-19 patient´s symptom severity. Despite our limited understanding of how HERVs are turned off upon infection clearance, or how HERVs mediate long-term effects when their transcription remains aberrantly on, the participation of these elements in neurologic disease, such as multiple sclerosis, is already settling the basis for effective therapeutic solutions. These observations support an urgent need to identify the mechanisms that lead to HERV expression with SARS-CoV-2 infection, on the one hand, and to answer whether persistent HERV expression exists in post-COVID-19 condition, on the other. The present study shows, for the first time, that the HERV-W ENV protein can still be actively expressed long after SARS-CoV-2 infection is resolved in post-COVID-19 condition patients. Moreover, increased anti-SARS-CoV-2 immunoglobulins in post-COVID-19 condition, particularly high anti-SARS-CoV-2 immunoglobulin levels of the E isotype (IgE), seem to strongly correlate with deteriorated patient physical function (r=-0.8057, p<0.01). These results indicate that HERV-W ENV antigenemia and anti-SARS-CoV-2 IgE serology should be further studied to better characterize post-COVID-19 condition pathogenic drivers potentially differing in subsets of patients with various symptoms. They also point out that such biomarkers may serve to design therapeutic options for precision medicine in post-COVID-19 condition.

## Introduction

The sanitary and economic burden associating with patient post-viral COVID-19 sequalae, months or even years after virus clearance constitutes a prominent pandemic aftermath challenge, particularly when considering the large number of affected individuals worldwide (1). Although this medical condition was listed in the ICD-10 as “post-COVID-19 condition” since September 2020 (2), the term popularized for SARS-CoV-2 post-viral syndrome whose main persistent symptoms include sore throat, dyspnea, chronic fatigue, pain, intestinal and sleeping disturbances, cognitive deficits, anxiety and depression (3) seems to be “long COVID-19”. Current clinical treatments for post-COVID-19 condition are merely symptom palliative since the potential mechanisms triggering or underlying the syndrome remain undefined and no specific clinical biomarkers have yet been identified (3, 4). A situation shared by other diseases of unknown etiology presenting overlapping symptomatology with post-COVID-19 (5–7).

The intense research in this field is, however, starting to yield valuable hints to improve our understanding of post-COVID-19 condition physiologic derangements, as for example the remodeling of T cell dynamics upon SARS-CoV-2 infection (8), the viral-induced autoimmunity (9, 10), or the harm derived from SARS-CoV-2 neuro-invasive capacity (11, 12). Viral infections may trigger epigenetic changes leading to human endogenous retrovirus (HERVs) aberrant expression and chronic innate immune activation which translates into patient post-viral symptoms (13).

HERVs are ancient retroviral DNA sequences integrated into the human genome during evolution by reaching the germline, which constitute 8% of its size (14). Despite the benefits provided by their regulatory and coding capacities (14–16), it is widely documented that aberrant HERV expression correlates with neurological disease (14, 17–19). The recent detection of the multiple sclerosis antigen HERV-W ENV in the blood of acute COVID-19 patients, and its demonstrated relationship with disease severity and inflammation (20, 21), raised the question on whether, similarly to Epstein-Barr Virus (EBV) infection and multiple sclerosis (22), SARS-CoV-2 infection may trigger long-term neurologic disease through HERV activation.

On another end, the study of antibody response to SARS-CoV-2 by screening virus antigen microarrays revealed that early IgA and IgG responses can constitute markers of acute disease severity (23). In addition, it was found that SARS-CoV-2 elicits IgE responses with levels positively correlating with severity thus suggesting a link between SARS-CoV-2 infection, degree of severity with mast cell activation (23, 24).

This study aimed at determining if in fact HERV-W ENV expression remains active in post-COVID-19 patients and, if so, what is the relationship between HERV-W ENV expression and anti-SARS-CoV-2 immunoglobulin levels, both being increased in acute SARS-CoV-2 infections with severity, in reference to patient symptoms. Importantly, the outcome could serve to unravel post-COVID-19 subjacent mechanisms relating to acute SARS-CoV-2 infections. Information holding diagnostic and therapeutic implications.

## Materials and methods

### Participating individuals and associated data

This cross-sectional study was approved by the Public Health Research Ethics Committee DGSP-CSISP of Valencia, Valencia, Spain, núm. 20210604/04/01. Samples and data from patients included in this study were provided by the IBSP-CV Biobank (PT17/0015/0017), integrated in the Spanish National Biobanks Network and in the Valencian Biobanking Network and they were processed following standard operating procedures with the appropriate approval of the Ethics and Scientific Committees (num. 20210604/04/02). Provision of samples of acute COVID-19 cases from the biobank of “Hospices Civils de Lyon” was approved by the ethical committee (Centre de Resource Biologique de Hospices Civiles de Lyon, Hôpital de la Croix-Rousse, Lyon France) and the French Ministry of Research for the constitution of a collection of COVID-19 biological samples and their session for the purpose of research (Autorisation N°: DC-2020-3919 and AC-2020-3918). Peripheral blood mononuclear cells (PBMC) from healthy donors (HBD) were obtained under established legal activity for the provision of blood-derived samples from the “Etablissement Français du Sang” (EFS) of Lyon (France). The study included a total of 66 patients diagnosed with acute COVID-19 (n=22), post-COVID-19 condition (n=12), chronic fatigue syndrome (CFS) (n=17) -a disease presenting overlapping symptoms with post-COVID-19 condition- (pre-pandemic cases/samples) (7), pre-pandemic healthy blood donors (ppHBD) (n=4) and similar healthy donors with samples obtained during the pandemic (pHBD) (n=11) (**Supplementary Table 1**). CFS patients were diagnosed using the Canadian (25) and/or International Consensus (26) criteria. Post-COVID-19 condition patients suffered the disease for at least six months. Acute COVID-19 patients were hospitalized with moderate to severe clinical status based on the clinical scale for COVID-19, recommended by the National Institute of Health of the USA guidelines (https://www.covid19treatmentguidelines.nih.gov/overview/clinical-spectrum/).

Patients with health problems other than CFS or COVID-19 were excluded from the study and individuals with any similar or related pathology were excluded from HBD groups. Written informed consent was obtained from all study participants, and CFS and post-COVID-19 patient health status was evaluated with the use of standardized questionnaires, including the FIQ case report form (27, 28), the MFI questionnaire (29), and the quality-of-life SF-36 instrument (30).

### Blood collection and plasma isolation

Each participant donated 10 mls of blood obtained by standard phlebotomy in K2EDTA (BD cat. 366643 or Vacuette cat. 455045) tubes. All blood samples were kept at RT and processed within 4 h. Tubes were centrifuged at 500 xg for 15 min to separate plasma from the remaining blood components, the upper phase was transferred to a clean tube and further centrifuged at 8000 xg for 10 min to remove platelets and debris. Poor platelet plasma was aliquoted in cryovials (0.5-1 ml/tube) and kept frozen at -80°C until use, as previously described (31).

### Detection of HERV-W envelope protein and SARS-CoV-2 antigen serology

For the detection of HERV-W ENV antigen in plasma and the quantification of its circulating soluble form, all analyses were performed according to the conditions provided in the patent published under ref. WO2019201908 (A1) and entitled “Method for the detection of the soluble hydrophilic oligomeric form of HERV-W envelope protein”, as previously described (32). The specific signal was expressed as the signal to noise (S/N) ratio, where the noise represents the mean+2SD of the background signals yielded by a panel of HBD samples. Both HERV-W ENV antigenemia and SARS-CoV-2 serology were performed by Simple Western technology according to immunoglobulin class. Samples of ppHBDs and CFS patients, were stored over a year in freezers at -80°C. Pandemic and acute and post-COVID-19 condition samples were stored in freezers at -80°C for less than a year. All samples were kept frozen at all times after their initial freezing until use (single freeze/thaw cycle before the immunoassay). Detailed description of for SARS-CoV-2 serological analyses with multiple antigens detection is provided as **Supplementary Figures 1**, **2**.

### Statistical analyses

All statistical analyses were performed using Prism (version 8.0; GraphPad Software, La Jolla, California). Continuous data are expressed as means ± SD, as indicated. Normal data distribution was assessed using the Shapiro-Wilk test, assuming p-values > 0.05. Statistical differences were determined using t-student or Mann-Whitney tests, depending on whether the data followed or not normal distributions, respectively. To establish group differences, statistical significance was set at *p*<0.05.

## Results

### Demographics and clinical characteristics of participants

A total of 66 subjects were evaluated in this study corresponding to ppHBDs, n=4; pHBDs, n=11; pre-pandemic CFS patients, n=17; acute COVID-19 patients, n=22; and post-COVID-19 condition patients, n=12. A summary of Total Fibromyalgia Impact Questionnaire (FIQ) (27, 28), Multi Fatigue Inventory (MFI) general fatigue (29) and quality of life Short-Form-36 Health Survey (SF-36) questionnaire (30) scores for CFS and post-COVID-19 condition patients is shown in **Table 1**. Differences between CFS and post-COVID-19 condition were only observed with questionnaire scores for total FIQ (*p<*0.05), MFI general fatigue (*p<*0.01), SF-36 general health (*p<*0.05) and mental health (*p<*0.05), while all other areas among the 17 tested (**Table 1**) showed no significant difference, thereby suggesting widely overlapping symptomatology across these two groups of patients. However, questionnaire scores differences indicated more intense pain as measured by total FIQ and worse mental health as determined by the SF-36 instrument for the CFS group, while worse fatigue as assessed by the MFI and worse general health (SF-36) were observed for the post-COVID-19 condition cases (**Table 1**).

**Table 1:** Patient health status assessment with FIQ, MFI and SF-36 questionnaires (27–30).

Questionnaire	Post-COVID-19 condition (n=12)					Pre-pandemic CFS (n=17)					
	Mean	SD	±	SE	Range	Mean	SD	±	SE	Range	P.value
**FIQ**											
Total FIQ	65.14	11.79	±	3.40	47.95 - 81.63	77.57	13.01	±	3.15	47.76 - 96.26	**0.014**
Function	4.87	1.20	±	0.35	2.64 - 6.27	5.90	1.87	±	0.45	1.65 - 9.24	0.104
Overall	7.75	1.42	±	0.41	4.29 - 8.58	7.75	2.57	±	0.62	2.86 - 10	0.633
Symptoms	6.20	3.47	±	1.00	1.32 - 10	7.23	3.17	±	0.77	1.43 - 10	0.412
**MFI**											
General Fatigue	17.33	2.67	±	0.77	13 - 20	13.65	3.41	±	0.83	7 - 20	**0.004**
Physical Fatigue	16.58	3.58	±	1.03	10 - 20	14.35	3.20	±	0.78	12 - 20	0.090
Reduced Activity	15.75	4.33	±	4.33	8 - 20	13.41	2.98	±	0.72	11 - 20	0.090
Reduced Motivation	14.58	4.21	±	1.22	8 - 20	12.53	2.72	±	0.66	8 - 19	0.121
Mental Fatigue	14.83	3.95	±	1.14	6 - 20	13.06	2.75	±	0.67	9 - 19	0.164
**SF-36**											
Physical Functioning (PF)	42.50	16.99	±	4.90	25 - 80	33.29	16.81	±	4.08	0 - 65	0.160
Role Physical (RP)	17.71	17.84	±	5.15	0 - 56.25	12.13	21.13	±	5.13	0 - 75	0.228
Bodily Pain (BP)	30.63	30.49	±	8.80	0 - 100	16.03	14.50	±	3.52	0 - 45	0.192
General Health (GH)	31.25	12.45	±	3.60	5- 50	20.59	13.45	±	3.26	0 - 45	**0.039**
Vitality (VT)	17.71	14.56	±	4.20	0 - 37.5	11.84	10.91	±	2.65	0 - 31.25	0.224
Social Functioning (SF)	27.08	21.87	±	6.31	0 - 50	26.47	25.48	±	6.18	0 - 75	0.947
Role Emotional (RE)	65.28	34.42	±	9.94	0 - 100	39.67	38.82	±	9.41	0 - 100	0.078
Mental Health (MH)	61.67	14.20	±	4.10	35 - 80	46.24	16.51	±	4.00	15 - 80	**0.014**

FIQ, Fibromyalgia Impact Questionnaire; MFI, Multi Fatigue Inventory; SF-36, Short-Form-36 Health Survey; SD, standard deviation; SE, standard error. Range refers to the possible values in the studied group. Statistical tests: t-test for variables with normal distribution and equal variances or Mann-Whitney for variables not normally distributed. Bold numbers indicate significant differences between the two patient groups compared.

### HERV-W ENV antigenemia in post-COVID-19 condition

**Figure 1:**
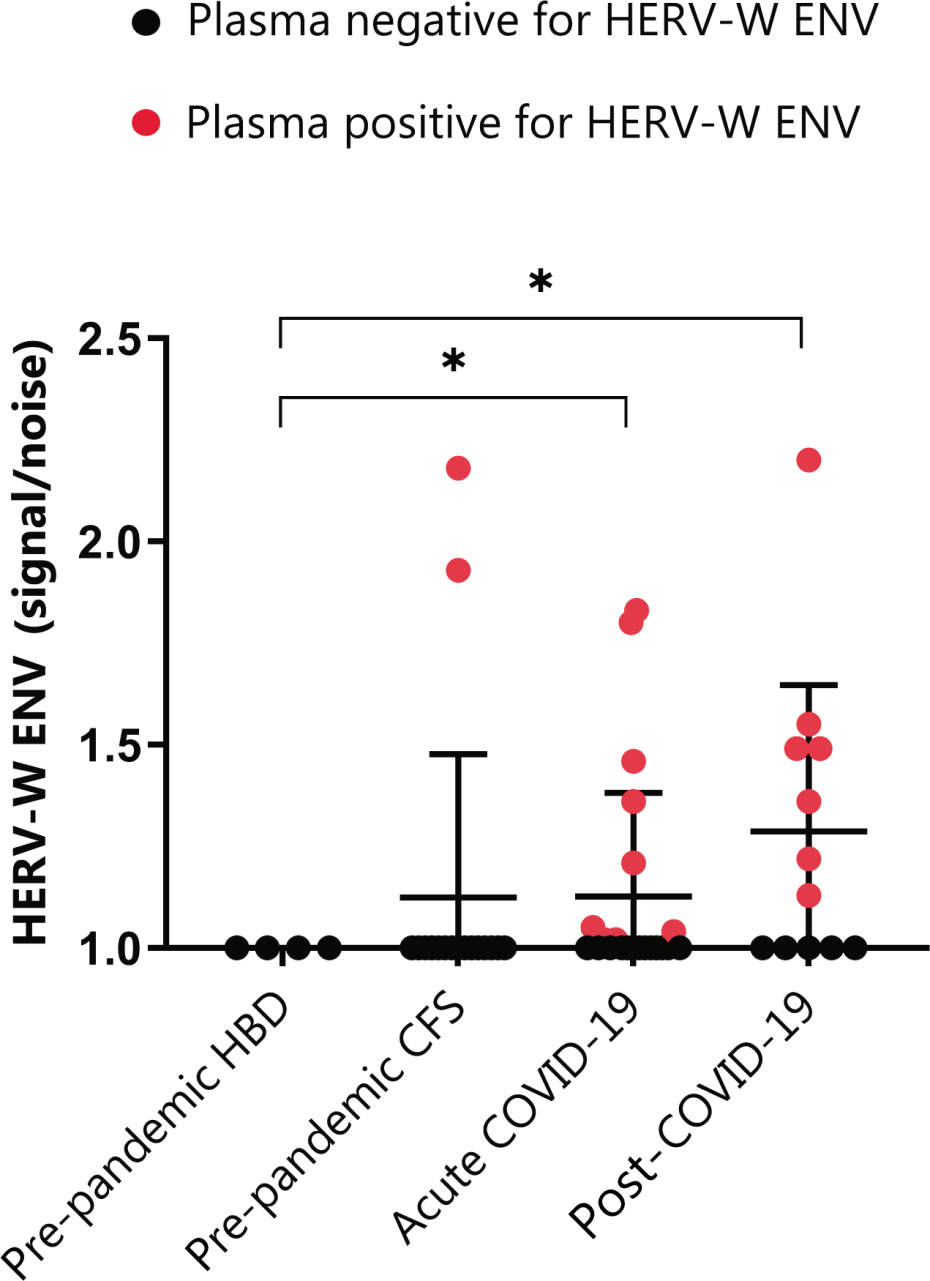
HERV-W envelope (ENV) antigenemia in pre-pandemic subjects (ppHBD, n=4), chronic fatigue syndrome cases (CFS, n=17), acute COVID-19 (n=22) and post-COVID-19 condition subjects (n=12). Levels of HERV-W ENV protein in plasma are shown normalized to noise (non HERV-W ENV specific signal). Samples positive for HERV-W ENV protein antigenemia are labeled in red. *p < 0.05, based on t-test with Welch’s correction.

Since previous studies had evidenced increased expression of the HERV-W ENV protein in acute COVID-19 patients in association with disease severity (20, 21), this study set to determine whether the presence of this protein remains high in post-COVID-19 patients. To this end, circulating levels of HERV-W ENV protein were measured in post-COVID-19 plasma in comparison to acute COVID-19, ppHBD, and pre-pandemic CFS subjects by Simple Western technology (**Figure 1**). The pre-pandemic CFS group was included as an additional control group sharing clinical symptoms with post-COVID-19 condition.

The analysis interestingly showed that a higher proportion of post-COVID-19 condition subjects, corresponding to 58% (7 out of 12), presented detectable HERV-W ENV protein as compared to only 41% (9 out of 22) of our acute COVID-19 cohort. Moreover, HERV-W ENV protein levels expressed as signal to noise (S/N) ratios were similar in post-COVID-19 condition patients (1.13-to-2.20) when compared to acute COVID-19 (1.02-to-1.8), but significantly different from all controls in both acute and post-COVID-19 condition cohorts (*p<*0.01). HERV-W ENV expression in long COVID included cases ranging from 6- to 19-month post-infection (**Supplementary Table 1**). Surprisingly, 2 out of 17 pre-pandemic CFS subjects were strongly positive (1.93 and 2.18) for HERV-W ENV expression, the significance of which is unknown at present.

### SARS-CoV-2 serology according to immunoglobulin class in post-COVID-19 condition

**Figure 2:**
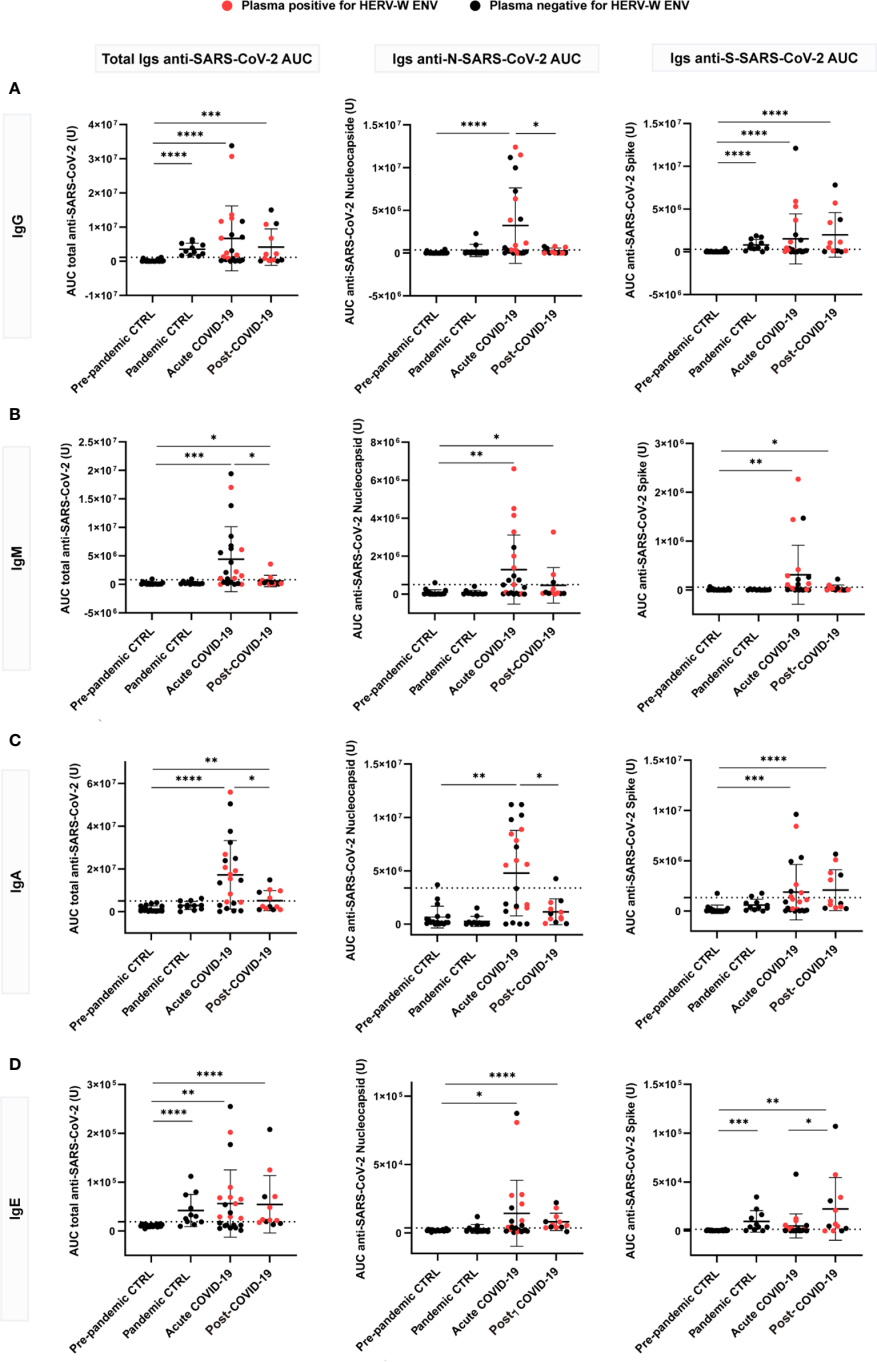
Anti-SARS-CoV-2 multi-isotype serology of acute (n=22) and post-COVID-19 condition (n=12) patients in comparison to pre-pandemic (n=21) and pandemic (n=11) control individuals. Levels of IgG **(A)**, IgM **(B)**, IgA **(C)** and IgE **(D)** against the SARS-CoV-2 nucleocapsid, spike or the combination of both (total) are shown as the mean of the area under the curve (AUC) according to electropherograms. Cut-off for each isotype was calculated as the mean of pre-pandemic controls + 3 standard deviations (SD). Samples positive for HERV-W ENV protein antigenemia are labeled in red. *p < 0.05, **p<0.01, ***p<0.001, ****p<0.0001 according to Mann-Whitney Test.

To explore the relationship of HERV-W ENV protein expression with patient immune response to SARS-CoV2 infection, we measured the levels of circulating anti-SARS-CoV-2 immunoglobulins (IgG, IgM, IgA and IgE) in acute and post-COVID-19 condition cases with respect to pre-pandemic (ppHBD + CFS) and pandemic (pHBD) control groups (also after vaccination campaign started) by Simple Western technology (**Figure 2**). The recombinant SARS-CoV-2 antigens present in the kit used to detect corresponding specific antibodies in human serum or plasma comprised the virus nucleocapsid and the spike proteins. As expected, pre-pandemic controls were negative for any of the anti-SARS-CoV-2 immunoglobulins while pandemic controls presented reactive IgG and, surprisingly, IgE to a certain extent for the spike antigen in the absence of reactivity to the nucleocapsid antigen, which should be consistent with vaccination (**Figures 2A, D**). Almost all individuals of this group (pHBD) had values over the cut-off for IgG and IgE against SARS-CoV-2 spike, which were statistically different in comparison to pre-pandemic controls (ppHBD + CFS) (*p<*0.0001 and *p<*0.001, respectively; right plots in **Figures 2A, D**), while very few IgA values and no IgM level surpassed the set cut-off values (right plots in **Figures 2B, C**).

However, the levels of anti-SARS-CoV-2 spike and nucleocapsid IgG, IgM, IgA and IgE appeared markedly increased in more than a half of acute COVID-19 cases in comparison to control groups (**Figure 2**). About 59% (13 out of 22) of the acute cases showed a significant increase of total (anti-spike + anti-nucleocapsid) IgG (*p<*0.0001), IgA (*p<*0.0001) and IgE (*p<*0.01) while increased total IgM were detected in even more cases (72%, 16 out of 22) (*p<*0.001) (**Figure 2**). This is consistent with a stage of active infection in which large amounts of IgM against the spike and nucleocapsid antigens are still generated. With respect to post-COVID-19 condition cases, a subgroup corresponding to 58% (7 out of 12) showed increased levels of anti-spike IgG compared to pre-pandemic controls (*p<*0.0001) (**Figure 2A**), and 42% (5 out of 12) showed increased anti-spike IgA levels (*p<*0.0001) (**Figure 2C**). These results do not seem to be related to recent vaccination, since subjects with equivalent elapsed time from vaccination present very different levels of those immunoglobulins (**Supplementary Figure 3**). Regarding IgM, just three post-COVID-19 condition cases showed increased anti-nucleocapsid IgM (**Figure 2B**). Strikingly, a large proportion of post-COVID-19 condition cases had detectable IgE levels against SARS-CoV-2 proteins (75%, 9 out of 12), which were significantly increased against the spike protein when compared to acute cases (p<0.05). Both anti-spike and anti-nucleocapsid IgE antibodies were significantly increased when compared to pre-pandemic controls (p<0.01 and p<0.0001, respectively; **Figure 2D**). These results highlight that a subgroup of post-COVID-19 condition patients present a sustained immune response against SARS-CoV-2 antigens, with IgG, IgA and IgE levels similar or even increased when compared to those observed during active COVID-19 infections (23, 24).

**Figure 3:**
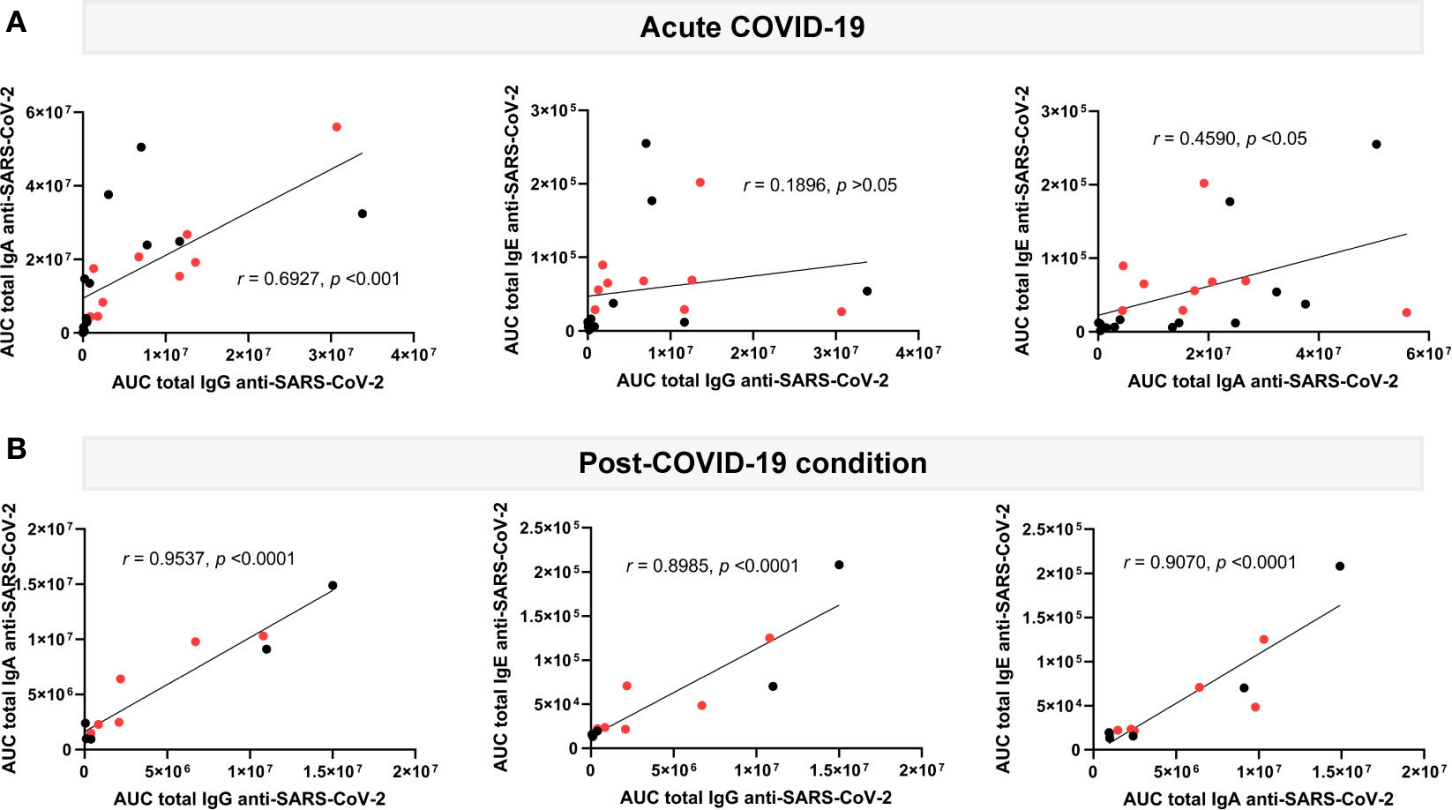
Correlation analysis of plasma circulating anti-SARS-CoV-2 immunoglobulin levels in acute (n=22) and post-COVID-19 condition (n=12). Levels of IgG, IgA and IgE against the SARS-CoV-2 nucleocapsid and spike (total) are shown as the mean of the area under the curve (AUC) according to electropherogram and its correlation, analyzed in acute **(A)** and post **(B)** COVID-19 samples by Pearson. Samples positive for HERV-W ENV protein antigenemia are labeled in red.

Immunoglobulin levels measured during acute COVID-19 presented moderate to no correlation with each other: IgG levels (**Figure 3A**, left and middle plots) showed a moderate correlation with IgA (*r=*0.6927, *p<*0.001), but none with IgE (*r=*0.1896, *p>*0.05), whereas showing a weak correlation between IgA and IgE levels (*r=*0.4590, *p<*0.05; **Figure 3A**, right plot). Quite differently, in post-COVID-19 condition, three out of the four immunoglobulins studied appeared strongly correlated (**Figure 3B**): positive correlations were found between IgG and IgA or IgE levels, which were close to 1 (*r=*0.9537, *p<*0.0001; and *r=*0.8985, *p<*0.0001, respectively; **Figure 3B**, left and middle plots). Likewise, IgA levels correlated with IgE levels to a high extent (*r=*0.9070, *p<*0.0001) (**Figure 3B**, right plot). This data supports the existence of a sustained immune response against SARS-CoV-2 antigens, consistent between all immunoglobulin isotypes but unexpectedly skewed towards IgE reactivity, months or even years after acute infection.

### Increased anti-SARS-CoV-2 immunoglobulins with HERV-W ENV

**Figure 4:**
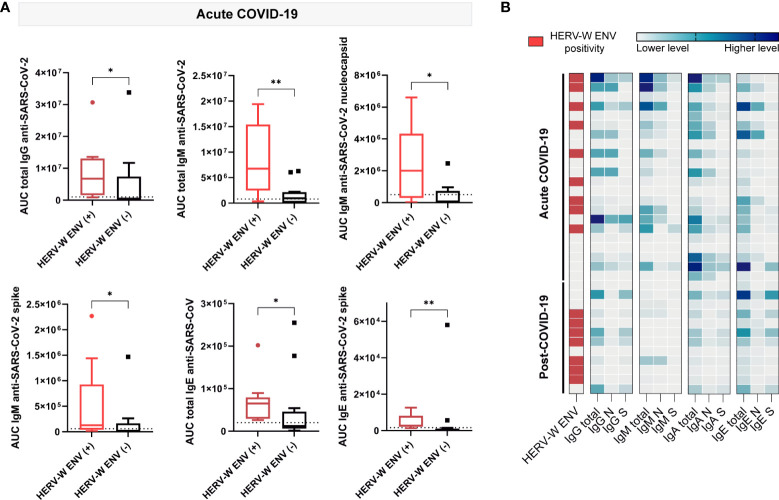
Comparison of plasma circulating anti-SARS-CoV-2 immunoglobulin levels in samples positive (+) and negative **(-)** for HERV-W ENV protein antigenemia. **(A)** Boxplots showing levels of IgG, IgM and IgE calculated as the mean of the area under the curve (AUC) according to electropherograms in positive and negative HERV-W ENV samples of acute COVID-19 patients. *p < 0.05, **p<0.01 according to Mann-Whitney Test. **(B)** Heatmap analysis of HERV-W ENV protein antigenemia and levels of IgG, IgM, IgA and IgE against SARS-CoV-2 nucleocapsid, spike or both (total) in acute (n=22) and post (n=12) COVID-19 cases.

To explore the potential relationship of SARS-CoV-2 antigenicity with the aberrant expression of HERV-W ENV protein, the levels of IgG, IgM, IgA and IgE against nucleocapsid or spike SARS-CoV-2 proteins were compared across plasma samples showing positive or negative presence of HERV-W ENV in acute and post-COVID-19 condition cases (n=22 and 12 respectively). Interestingly enough, several anti-SARS-CoV-2 immunoglobulins showed significant differences in accordance to HERV-W ENV antigenemia in the acute COVID-19 group (**Figure 4**). Samples positive for HERV-W ENV (n=9) had increased IgG (*p*<0.05), IgM (total: *p*<0.01, nucleocapsid: *p*<0.05) and IgE (total: p<0.05, spike: *p*<0.01) levels when compared to negative samples (n=13) (**Figure 4A**). However, no significant difference in SARS-CoV-2 immunoglobulin levels was found in accordance with HERV-W expression in the post-COVID-19 condition group, which, however, may relate to the low number of samples in this group (total n=12, positive n=7). A heatmap illustrating individual differences of the studied cohort is shown on **Figure 4B**. Though significant differences in specific immunoglobulin levels were observed in accordance with HERV-W ENV positivity in acute COVID-19 cases (**Figure 4A**), no specific correlation between HERV-W ENV and anti-SARS-CoV-2 immunoglobulins levels was found (data not shown).

### Correlation of anti-SARS-CoV-2 immunoglobulins with post-COVID-19 condition symptoms

**Figure 5:**
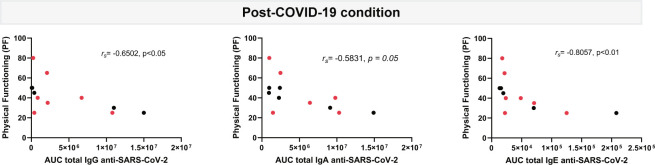
Correlation analysis of plasma circulating anti-SARS-CoV-2 IgG, IgA and IgE levels with physical functioning score from SF-36 questionnaire. Levels of IgG, IgA and IgE against the SARS-CoV-2 nucleocapsid and spike (total) are shown as the mean of the area under the curve (AUC) according to electropherograms and its correlation analyzed in post-COVID-19 condition (n=12) samples by Spearman. Samples positive for HERV-W ENV protein antigenemia are labeled in red.

To further evaluate the potential co-clustering of anti-SARS-CoV-2 immunoglobulin profiles with post-COVID-19 condition symptoms, we measured correlations between anti-SARS-CoV-2 immunoglobulins and patient scores with FIQ, MFI and SF-36 instruments assessing patient health status (**Figure 5**).

Although no significant correlations were found between the majority of the immunoglobulins and the questionnaire scores (data not shown), a moderate to strong correlation was associated with patient´s physical functioning, a variable assessing physical patient performance in the SF-36 questionnaire. As it can be observed in **Figure 5**, higher IgG (*r_s_=*-0.6502, *p<*0.05), IgA (*r_s_=*-0.5831, *p=*0.05) but, markedly, IgE (*r_s_=*-0.8057, *p<*0.01) levels associate with lower physical functioning scores relating to increased patient´s disability.

## Discussion

The initial anecdotal cases of COVID-19 long-haulers, term used to designate patients who developed long-lasting COVID symptoms, such as the famous case of a Portland 47-year-old woman who suffered COVID-triggered fever for almost a year (33), turned into a problem of high global proportions (49% affected over 120 days, as determined by Chen et al., recent systematic review and meta-analysis) (34). Clinicians soon realized that no relationship between severity and post-COVID-19 condition forecasting could be established as mild cases could become chronic while some intensive care patients completely returned to normality after a two-month recovery period (33). Among the patients affected on the long-term, two groups have been clearly differentiated based on organ (particularly lungs) damage or on the absence of evidence for such damage. The latter group being identified as potential developers of a syndrome comparable to myalgic encephalomyelitis/chronic fatigue syndrome (CFS) (7, 35), a severely disabling condition characterized by immuno-metabolic deficiencies and cognitive impairment lasting for many years (25, 26, 36). Thus, CFS previously reported to emerge after Lyme’s disease, mononucleosis, influenza, SARS or other infections (37–39), with an estimated worldwide prevalence of 0.89% (with 1.5 to 2-fold of women over men) with data gathered before 2020 (40), could acquire unprecedented values with catastrophic sanitary and economic consequences. This social awareness has motivated great concern and motivation to predict, resolve and prevent post-COVID-19 condition and CFS.

A common factor derived from epigenetic changes triggered by infections in neurologic disease is the activation of quiescent retroviral sequences present in the human genomes (HERVs) (13, 14). The finding that HERV-W ENV protein expression is increased in COVID-19 lymphocytes and the fact that HERV-W ENV levels correlate with pneumonia severity (20) motivated our search to determine whether these sequences remain unsilenced in post-COVID-19 condition, perhaps constituting a potential trigger or enhancer for certain symptoms. Our results (**Figure 1**) evidence for the first time the presence of the HERV-W ENV protein in about one-half of the analyzed plasma samples from post-COVID-19 condition patients. Despite the limited number of long COVID cases analyzed (n=12), the higher proportion and values for HERV-W ENV expression when compared to acute COVID-19 cases (n=22) (58% and 1.13 to 2.20 *vs.*,41% and 1.02 to 1.8) suggests a potential association of this HERV-encoded protein with the post-COVID-19 condition or, more likely, with a sub-group of patients with related pathogenic consequences. In cases from the present cohort, HERV-W ENV antigenemia is also seen to persist over one-year post-infection (**Supplementary Table 1**) suggesting that chronic expression is made possible after COVID-19 as seen to be lifelong in multiple sclerosis. Indeed, long-COVID-19, or post-COVID, obviously represents a heterogeneous nosological entity, despite common or overlapping symptomatology between patients (41–43).

A mechanism that may explain or favor the activation of HERV-W ENV expression by SARS-CoV-2 could be promoter binding site enrichment for transcription factors involved in immune responses. This would be consistent with the present findings of a correlation between specific immunoglobulin responses and HERV-W ENV expression in acute COVID-19, as well as with the persisting but peculiar humoral immunity in post-COVID-19 condition. While further overexpression of HERV-W ENV may also result from unspecific activation of NF-kB by different viral responses (44), transcription from specific transposable elements (TEs) may be viral agent- and cell phenotype-specific, according to the differential expression obtained from cell cultures infected with Middle East respiratory syndrome coronavirus (MERS-CoV or MERS), influenza A virus (IAV), respiratory syncytial virus (RSV), human parainfluenza virus type 3 (HPIV3), SARS or SARS-CoV-2 (45). Therefore, the study of specific TE profiles altered by SARS-CoV-2 may also reveal relevant features of the deranged cellular pathways in the immune cells of post-COVID-19 condition patients.

Interestingly, the highest HERV-W ENV values were obtained from two pre-pandemic CFS cases analyzed here as control samples (not post-COVID-19 condition but presenting overlapping symptoms) (**Figure 1**). The CFS results support previous findings showing HERV activation in CFS at the transcriptional level (31, 46) and demand further interrogation of HERV element expression at both transcriptional and protein levels. Whether HERV-W ENV aberrant expression is a common aspect to CFS and post-COVID-19 condition, or perhaps other post-viral syndromes, is unknown at present. Of note, it may also define subgroups with related etiopathogenesis in such clinically-defined conditions, as already found in psychiatric disorders with characterized immune inflammation (32).

In an effort to further explore the relationship between the activation of the immune system by SARS-CoV-2 and HERV-W ENV expression the exact same samples tested for HERV-W ENV expression were assayed for SARS-CoV-2 multi-isotypes serology (as total anti-SARS-CoV-2 antigens or directed to either SARS-CoV-2 nucleocapsid or spike proteins). **Figure 2** shows that SARS-CoV-2 antibody response is increased in acute COVID-19 cases but, rather unusually, for all isotypes (IgG, IgM, IgA and IgE); post-COVID-19 condition cases still had significant levels of anti-SARS-CoV-2 spike IgG, IgA and remarkably above acute cases, IgE isotype, but not of the IgM isotype. The absence of IgM is consistent with a post-acute period, but individual cases still had anti-N SARS-CoV-2 IgM, which should be considered on larger series since gastrointestinal persistence of SARS-CoV-2 infection has now been reported in long COVID cases (47). This also appears consistent with the still elevated anti-spike IgA levels in a significant number of patients with long COVID. Globally, the levels of anti-SARS-CoV-2 spike IgG and IgA observed in the post-COVID-19 condition group are significantly beyond those observed in pre-pandemic healthy controls and correlated to each other as well as to also significantly elevated IgE levels (**Figure 3B**).

Two parameters appear to emerge from this pilot study, beyond a mere interest as biomarkers, as potential contributors in the pathogenic pathways involved in long/post-COVID: (i) HERV-W ENV, known to be a potent TLR4 agonist in immunopathogenic as well as in neurotoxic processes (48–50), and (ii) IgE antibodies known to be strong effectors of immunoallergic conditions as well as anti-parasite humoral responses (51, 52). Thus, the present study strongly suggests focussing further studies on their potential role in certain forms and/or symptoms of post-COVID syndrome.

Our post-COVID-19 condition patients also presented increased anti-nucleocapsid IgE antibodies. Although the significance of this finding is unknown at present, it seems worth mentioning the correlation found by Matyushkina et al. between anti-SARS-CoV-2 nucleocapsid reactivity and autoimmunity in long COVID patients (10).

COVID-19 vaccination intends to boost anti-SARS-CoV-2 spike antibodies (53). In fact, induction of IgG and IgA have been observed with COVID-19 mRNA vaccination with similar induction kinetics, but faster IgA decline after 100 days (54). The absence of IgM anti-SARS-CoV-2 spike protein in control and post-COVID-19 condition participants could be easily explained in the absence of recent infection or vaccination in this group (**Supplementary Table 1**).

To further determine whether SARS-CoV-2 serological profile holds any relationship with HERV expression in acute or post-COVID-19 condition cases, we stratified patient´s according to HERV-W ENV positive or negative expression, and then compared SARS-CoV-2 antibody responses in each subgroup. All anti-SARS-CoV-2 immunoglobulins analyzed showed increased levels with HERV-W ENV expression in acute COVID-19 cases, with the exception of IgA (**Figure 4**), an observation that may explain the more severe evolution and symptoms already reported in this subgroup of acute COVID-19 patients (20, 21). No differential SARS-CoV-2 IgG, IgM and IgA profile could be attributed to the presence of HERV-W ENV in post-COVID-19 condition but a positive trend was observed for anti-S IgE. Whatsoever, the low number of post-COVID-19 condition cases positive for HERV-W ENV (n=7) in this small pilot cohort does not allow to perform accurate statistical comparisons.

Finally, one aim of this analysis of anti-SARS-CoV-2 humoral immunity was to determine whether a sustained immune response or a given isotype profile could characterize post-COVID-19 condition patients. Interestingly, the physical functioning scores, as measured with the standardized, validated, SF-36 instrument (30) appeared significantly correlated with higher Ig levels, particularly IgE levels (*r_s_=*-0.8057, *p<*0.01), meaning that these post-COVID-19 condition patients with high anti-SARS-CoV-2 IgE antibodies had related impaired ability to perform basic activities of daily living (**Figure 5**). This raises the possibility that a derangement of patient´s immune system is an underlying cause of, and/or that such a serological profile is reflecting long COVID patients compromised health. This study highlights an abnormal response to a virus with an IgE isotype, normally seen in parasitic infections or immunoallergic reactions, providing an additional indication that SARS-CoV-2 induces peculiar immune reactions and is likely to dysregulate both cellular and humoral immunity. In fact, it is already known that SARS-CoV-2 infection involves lymphocyte impairment leading to lymphopenia in severe acute cases, frequent hyperneutrophilia, and hyperactivation of innate-immunity (55–57).

However, the antibody detection appears to decrease to the limits of detection over time as seen with the post-infection and post-vaccination delays, which may limit such serological analyses and correlations to several months after SARS-CoV-2 infection or would require a more sensitive technique to detect these antibodies on a longer period. Anyhow, long-COVID (or post-COVID) diagnostic and therapeutic intervention, when made possible on the bases of precision medicine for this rather heterogeneous nosological entity, should take place within this relatively early period and not too late or when chronicity of symptoms has been established with modifications in the pattern of biomarkers, which may then create difficulties to establish a link with previous SARS-CoV-2 infection. A limiting aspect that quite likely has impacted the study of CFS and other post-viral syndromes. The present pilot results therefore suggest that precision medicine becomes possible for such disease conditions, which future studies should elaborate.

After this cross-sectional study has been performed for a pilot evaluation, studies with larger series are foreseen to include a longitudinal analysis to analyze the level of expression of both HERV-W ENV and IgE proteins and certain clinical characteristics/symptoms of long/post- COVID-19. This could however not be envisaged and set up without the rationale now provided by these first results.

## Data availability statement

The original contributions presented in the study are included in the article/**Supplementary Material**. Further inquiries can be directed to the corresponding authors.

## Ethics statement

The studies involving human participants were reviewed and approved by Dirección General de Salud Pública-Centro Superior de Investigación en Salud Pública (DGSP-CSISP) of Valencia, Valencia, Spain, núm. 20210604/04/01 and Centre de Resource Biologique de Hospices Civiles de Lyon, Hôpital de la Croix-Rousse, Lyon France, Autorisation N°: DC-2020-3919 and AC-2020-3918. The patients/participants provided their written informed consent to participate in this study.

## Author contributions

KG-O: data analysis, investigation, data curation, figure drawing, writing original draft and manuscript review. JP: serological and antigenemia analyses; graphs, figures drawing and manuscript review. JB: serological and antigenemia analyses; graphs, figures drawing and manuscript review. BC: methodology, management interpretation of analyses and manuscript review. EM-M: patient diagnosis, data gathering and manuscript review. HP and EO: conceptualization, funding acquisition, supervision, formal analysis, investigation, data curation, writing original draft and manuscript review. All authors contributed to the article and approved the submitted version.

## Funding

This study was funded by an Star Exclusivas SL grant, an ME Research UK (SCIO charity number SC036942) grant and by Generalitat valenciana CIAICO/2021/103 grant to EO. KG-O is supported by the Generalitat Valenciana ACIF2021/179 grant. Funders were not involved in any of the research stages.

## Acknowledgments

We want to particularly acknowledge the patients and the IBSP-CV Biobank (PT17/0015/0017) integrated in the Spanish National Biobanks Network for their collaboration. Authors also want to express their gratitude to Dr. Vicente Serra (Umivale, Valencia, Spain) for his help in the recruitment of volunteers.

## Conflict of interest

Authors JP, JB, BC and HP were employed by company GeNeuro-Innovation. HP, BC, JB and BC receive compensation from GeNeuro-Innovation for their work.

The remaining authors declare that the research was conducted in the absence of any commercial or financial relationships that could be construed as a potential conflict of interest.

## Publisher’s note

All claims expressed in this article are solely those of the authors and do not necessarily represent those of their affiliated organizations, or those of the publisher, the editors and the reviewers. Any product that may be evaluated in this article, or claim that may be made by its manufacturer, is not guaranteed or endorsed by the publisher.
