# Supplementary material for: HERV-W ENV antigenemia and correlation of increased anti-SARS-CoV-2 immunoglobulin levels with post-COVID-19 symptoms
Source: Front Immunol. 2022 Oct 27;13:1020064. doi: 10.3389/fimmu.2022.1020064 (PMC9647063; doi:10.3389/fimmu.2022.1020064)
Supplement: Supplementary file 1 [file DataSheet_1.pdf]

frontiers

Supplementary Material

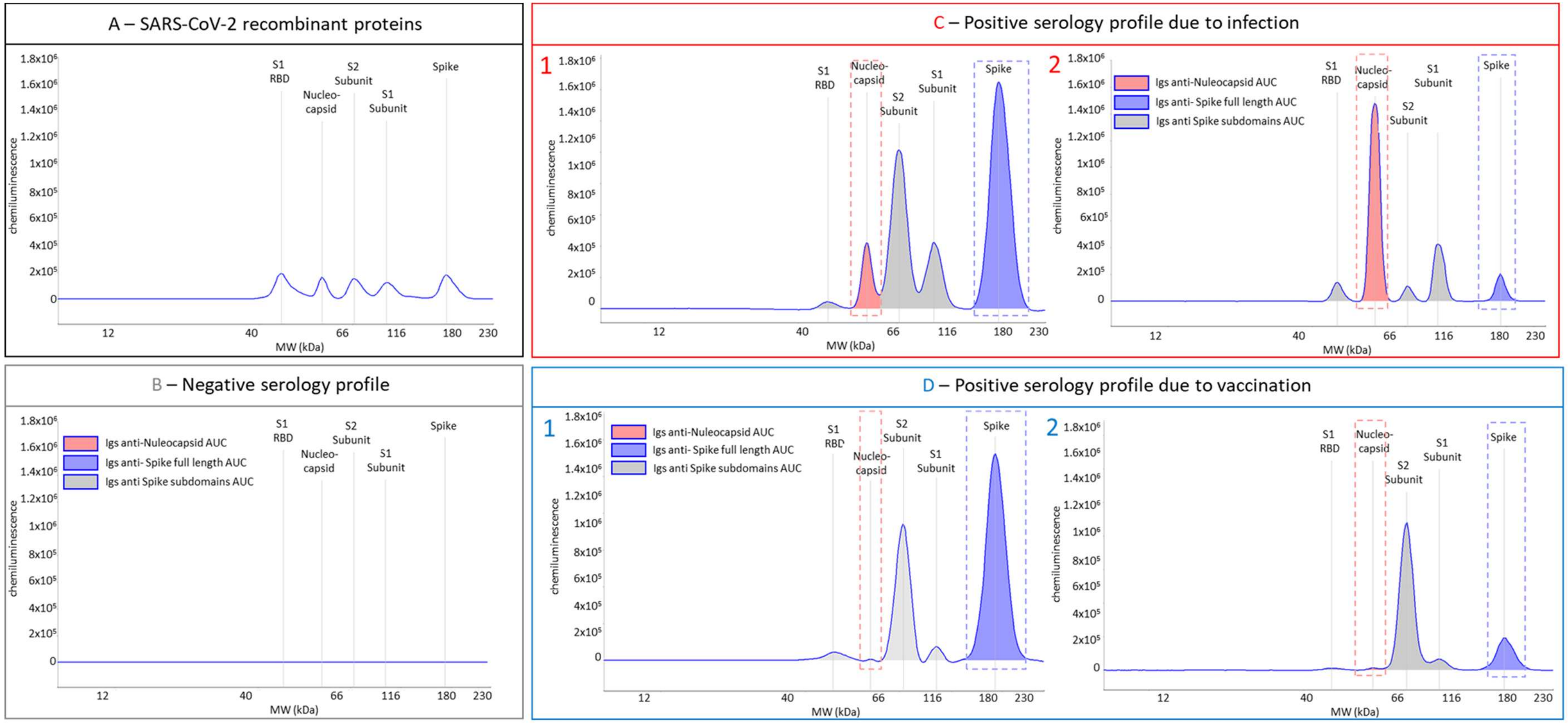

Supplementary Figure 1. Example of patient serology profile using Simple Western technology: A, Electropherogram using anti-his-tag antibody revealing the position (molecular weight) of his-tagged recombinant proteins of SARS-CoV-2 bound on capillary wall. B to D2, Representative electropherograms of sera with negative serology for SARS-CoV-2 (B; Igs anti-Nucleocapsid - /Igs anti-Spike -), a positive serology conferred by SARS-CoV-2 infection (two profiles presented here, C1 and C2, Igs anti-Nucleocapsid + /Igs anti-Spike +) and a positive serology conferred by vaccination (two profiles presented here, D1 and D2, Igs anti-Nucleocapsid - /Igs anti-Spike +). A to B4, Clear red, clear blue and grey area under

curve respectively correspond to the quantified AUC of Nucleocapsid (clear red dotted square), full length Spike (clear blue dotted square) and Spike subunits.

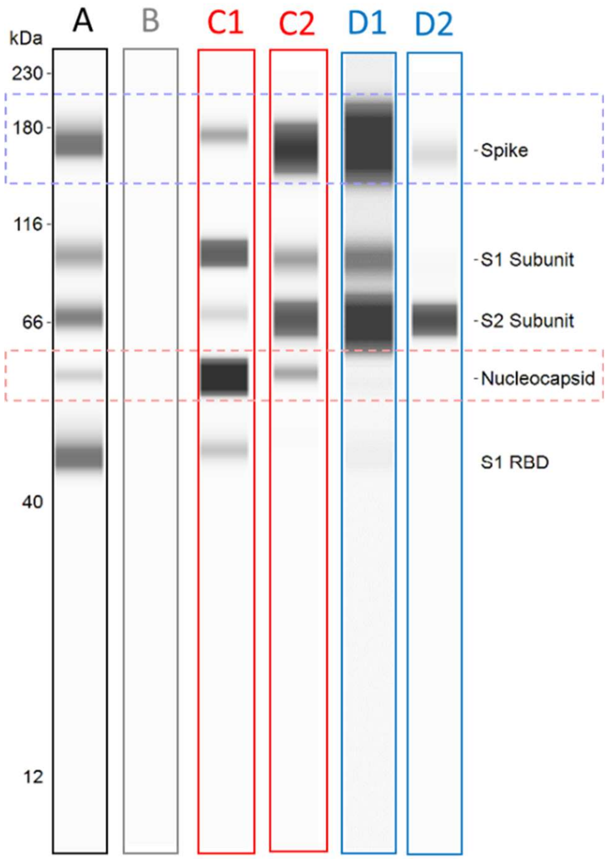

Supplementary Figure 2. Digital Western blot profiles constructed from electropherograms: Digital Western blots, constructed from the AUC value of each peak recorded on electrophograms (see Figure Y), have been provided from A to D2. The intensity of the band is proportional to the AUC of the peak associated with it. The clear red and clear blue dotted squares correspond to Nucleocapsid and full length Spike specific bands respectively.

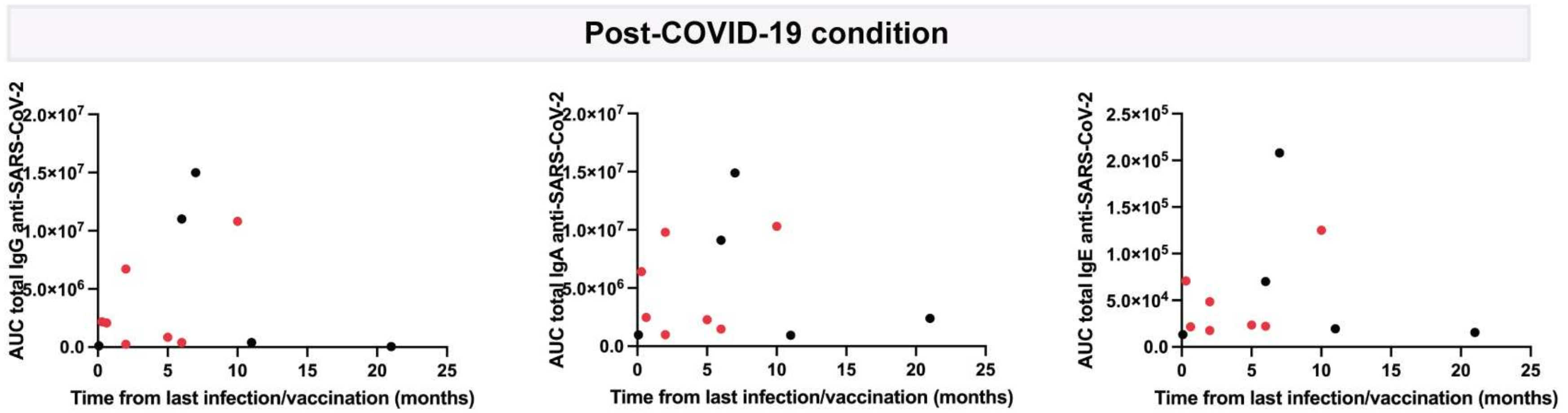

Supplementary Figure 3. Scatter plot of IgG, IgA or IgE levels (y axis) and time elapsed since last vaccination/infection (x axis) in post-COVID-19 patients (n=12).
